# Profile of People living with Dementia Referred to Neuropalliative Care Clinic at a Tertiary Care Center

**DOI:** 10.1002/alz70858_102654

**Published:** 2025-12-26

**Authors:** Dinesh Kumar B, Priya Treesa Thomas, Faheem Arshad, Suvarna Alladi

**Affiliations:** ^1^ NIMHANS, Bengaluru, Karnataka, India; ^2^ National Institute of Mental Health and Neurosciences [NIMHANS], Bengaluru, Karnataka, India; ^3^ National Institute of Mental Health and Neurosciences, Bengaluru, Karnataka, India

## Abstract

**Background:**

Dementia is a neurodegenerative condition that progressively impairs cognitive and functional abilities, significantly affecting the quality of life. People living with dementia (PLWD) will benefit from timely referral to Neuropalliative care (NPC) services in enhancing symptom management patient comfort and provide support for caregivers. Understanding the profile of people living with dementia referred to these services is essential for developing personalized care strategies. The present study aimed to explore the profile of people living with dementia referred to NPC and assess the severity of disease by using Global deterioration scale.

**Materials and Methods:**

A cross sectional study was conducted among the people registered in the department of Neurology of a tertiary referral care center for Neuropsychiatry and referred to a Neuropalliative care clinic. The participants who consented for the study were interviewed with a predetermined proforma to collect clinical parameters and variables such as age, gender, marital status, education level, socio‐economic status, and comorbid conditions. Global deterioration Scale was used to assess the disease severity by the patients in terms of objective and subjective. Descriptive statistics were used to analyze the data.

**Results:**

A total of 216 dementia patients were received to Neuropalliative care services during the study period. (*n* = 75) had dementia, followed by dementia subtypes (*n* = 92), dementia with overlap syndromes (*n* = 49). Mean age of patients was 61, SD 9.90. with 42.1% being male and 57.9% female. 85.2% of patients were married, while 12.5% were widowed. The majority (*n* = 63) had a secondary level of education. Socio‐economic status was predominantly BPL (*n* = 148) Category, and most common comorbidities was diabetes. Patients had an average score of 5.2, SD 1.24 in the Global deterioration Scale, indicating a moderate level of cognitive decline.

**Conclusion:**

The study highlighted important factors that may influence care planning and the need for targeted interventions. Future research will focus on longitudinal outcomes to evaluate the effectiveness of Neuropalliative care in people with dementia.

